# Supplementary material for: Conditional chemoconnectomics (cCCTomics) as a strategy for efficient and conditional targeting of chemical transmission
Source: eLife. 2024 Apr 30;12:RP91927. doi: 10.7554/eLife.91927 (PMC11060718; doi:10.7554/eLife.91927)
Supplement: Figure 3—source data 3. [file elife-91927-fig3-data3.zip › Figure 3 source data3 part 2.zip!/Figure 3-Source data 3-part2/1053f3-1-CRISPResso_Report_t6djfp6u.zip!/CRISPResso_on_1053f3_2/9.Alleles_frequency_table_around_sgRNA_ggccaccagatgcaaaatcg.pdf]

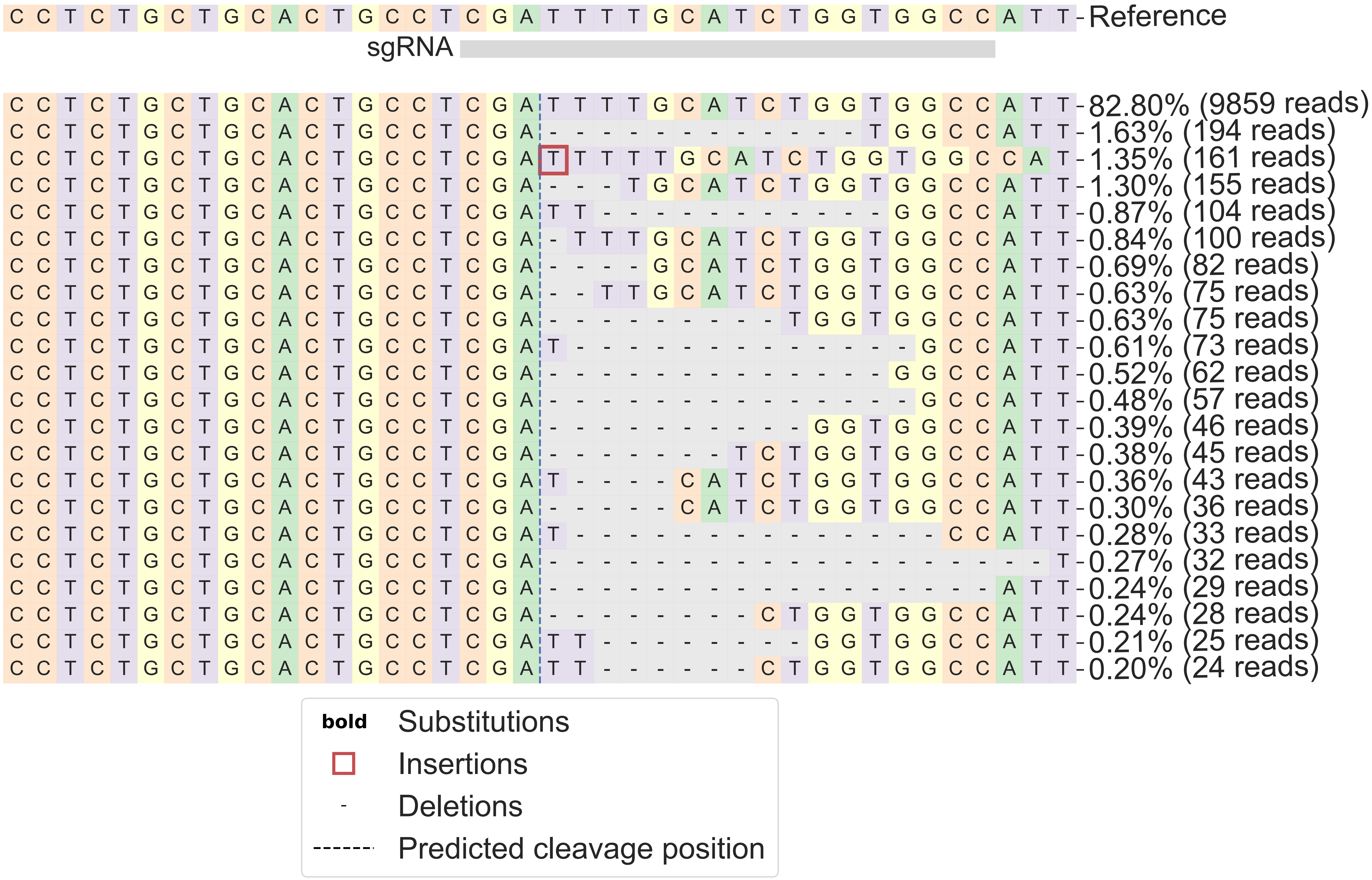

CCTCTGCTGCACTGCCTCGATTTTGCATCTGGTGGCCATT
Reference
sgRNA
82.80% (9859 reads)
1.63% (194 reads)
1.35% (161 reads)
1.30% (155 reads)
0.87% (104 reads)
0.84% (100 reads)
0.69% (82 reads)
0.63% (75 reads)
0.63% (75 reads)
0.61% (73 reads)
0.52% (62 reads)
0.48% (57 reads)
0.39% (46 reads)
0.38% (45 reads)
0.36% (43 reads)
0.30% (36 reads)
0.28% (33 reads)
0.27% (32 reads)
0.24% (29 reads)
0.24% (28 reads)
0.21% (25 reads)
0.20% (24 reads)
bold Substitutions
Insertions
Deletions
Predicted cleavage position
